# Amount of health care and self-care following a randomized clinical trial comparing flexion-distraction with exercise program for chronic low back pain

**DOI:** 10.1186/1746-1340-14-19

**Published:** 2006-08-24

**Authors:** Jerrilyn A Cambron, M Ram Gudavalli, Marion McGregor, James Jedlicka, Michael Keenum, Alexander J Ghanayem, Avinash G Patwardhan, Sylvia E Furner

**Affiliations:** 1 Department of Research, National University of Health Sciences, Lombard IL, USA; 2 Division of Epidemiology and Biostatistics, School of Public Health, University of Illinois at Chicago, USA; 3 Palmer Center for Chiropractic Research, Palmer College of Chiropractic, Davenport IA, USA; 4 Independent Consultant, Plano TX, USA; 5 Department of Chiropractic Practice, National University of Health Sciences, Lombard IL, USA; 6 Orthosport Physical Therapy, Inc., Chicago IL, USA; 7 Department of Orthopaedic Surgery and Rehabilitation, Loyola University- Stritch School of Medicine, Maywood IL, USA; 8 Edward Hines Jr. Veterans Affairs Hospital, Hines IL, USA

## Abstract

**Background:**

Previous clinical trials have assessed the percentage of participants who utilized further health care after a period of conservative care for low back pain, however no chiropractic clinical trial has determined the total amount of care during this time and any differences based on assigned treatment group. The objective of this clinical trial follow-up was to assess if there was a difference in the total number of office visits for low back pain over one year after a four week clinical trial of either a form of physical therapy (Exercise Program) or a form of chiropractic care (Flexion Distraction) for chronic low back pain.

**Methods:**

In this randomized clinical trial follow up study, 195 participants were followed for one year after a four-week period of either a form of chiropractic care (FD) or a form of physical therapy (EP). Weekly structured telephone interview questions regarded visitation of various health care practitioners and the practice of self-care for low back pain.

**Results:**

Participants in the physical therapy group demonstrated on average significantly more visits to any health care provider and to a general practitioner during the year after trial care (p < 0.05). No group differences were noted in the number of visits to a chiropractor or physical therapist. Self-care was initiated by nearly every participant in both groups.

**Conclusion:**

During a one-year follow-up, participants previously randomized to physical therapy attended significantly more health care visits than those participants who received chiropractic care.

## Background

People impaired with back pain frequently seek help from medical professionals. In 1999, there were about 15 million office visits to physicians in the U.S. for low back pain, accounting for about 2.8% of all office visits. Because this number did not include visits to other health care professionals, such as chiropractors, the actual number of office visits was probably more than 30 million per year [1]. Health care expenditures related to back pain reached $26.3 billion in 1998 in the United States alone [2]. Feuerstein et al. assessed the 1997 National Medical Expenditure Panel Survey, and determined that of the participants with low back pain, the majority sought medical management (73.7%), chiropractic care (30.6%), or physical therapy (9.3%) [3]. Within this low back pain population, the average number of visits per year was 3.8 medical visits, 7.8 chiropractic visits, and 8.4 physical therapy visits [3]. These results give us an idea of the health care utilized by individuals in the general population who suffer with low back pain.

Care seeking behavior by patients with low back pain is most commonly associated with increased pain and disability [4-6], meaning more care is sought when worse symptoms are experienced. The amount of health care utilized may therefore be used as a measure of patient health status, and thus may be compared between groups of patients to determine effectiveness of certain therapies. The purpose of this study is to assess if there is a difference in the total annual number of office visits for low back pain after a four-week clinical trial of either chiropractic care (flexion distraction) or physical therapy (exercise program) for treatment of chronic low back pain.

Proctor et al. determined that about 25% of patients with chronic, disabling, work-related musculoskeletal disorders pursue new health care services after completing a course of treatment, and among those who sought additional health care from a new provider, a subgroup of <15% (3.7% of the entire cohort) accounted for a disproportionate share of lost worker productivity, more surgical procedures, and ongoing financial disputes [7]. They further stated that in patients with chronic, disabling, work-related musculoskeletal disorders, post-treatment utilization of health care from a new provider is an important dimension of outcome, suggesting that categories to be measured should include: (1) the percentage of patients seeking care from a new provider, (2) the number of visits to the new provider over and above visits with the health care professionals overseeing all treatment, and (3) new surgery at the involved anatomic area or areas.

A few investigators have measured health care utilization following participation in a clinical trial on low back pain. Mayer et al. completed a randomized clinical trial comparing a rehabilitation and pain management program for low back pain with a no-treatment group [8]. During the one-year follow-up, these investigators determined that additional surgery rates were comparable for both groups (6% in the treatment and 7% in the no treatment group). However, the percentage of participants who sought additional health care was substantially lower in the treatment group (29%) compared to the comparison group (56%). The average numbers of total visits to health care professionals during the year of follow-up were also substantially different with an annual average of 1.6 visits in the treatment group versus 17.1 visits in the comparison group.

Similarly, Bendix et al. found a significant difference in the number of health care visits in participants with chronic low back pain during the year following randomization to either a 3-week intensive functional restoration program versus a less intense 8-week physical training program [9-12]. The average annual number of contacts with family doctors, chiropractors, physical therapists, and other health-care workers combined was significantly lower in the functional restoration program (2.5 visits) versus the physical training program (4.0 visits). These authors completed a parallel study also on patients with chronic low back pain; however participants in this study were randomized to three different groups, including a functional restoration program, an active physical training and back school, or psychological pain management and active physical training [9-11]. After one year, participants in this study also had a significantly different average number of health care contacts (4.5, 11.8, and 12.0 respectively) demonstrating a greater need for care in the latter two groups [9].

Not all investigators have observed group differences in post-treatment health care utilization. Berwick et al. randomized participants to three types of conservative care for low back pain, including usual care, back school, or back school with a self management component, and then followed the participants for one year [13]. In this study, the percent of participants who visited the primary care provider for back pain during the one-year follow-up was not significantly different (38%, 38%, and 42% respectively), nor were the average number of visits per year (1.03, 1.13, and 1.62 respectively).

One study went beyond measuring the aggregate number of office visits, and separated the visits based on provider type. Goossens et al. compared three conservative care methods for treatment of chronic low back pain then followed the participants for one year [14]. During the year of follow-up, participants who previously received rehabilitation with individual psychotherapy visited a general practitioner an average of 7.0 times, participants in the rehabilitation with group psychotherapy visited a general practitioner an average of 7.9 times, and participants who received rehabilitation only visited the general practitioner 6.0 times. Visits to "specialists" (5.1, 4.6, and 2.8 respectively), to physiotherapists (21.5, 12.9, and 10.6 respectively), and to alternative medicine practitioners (1.6, 1.0, and 5.5 respectfully) were appreciably different. However, no statistical analyses were performed on these measures to determine significance.

Various clinical trials on chiropractic care for low back pain have tracked the use or non-use of health care during follow-up studies, and a portion of all treatment groups have been found to seek further care [15-19]. Other studies have also tracked the amount of health care utilized outside of a clinical trial. However, no investigator has determined the *amount *of health care utilized for back pain after participation in a clinical trial on chiropractic care. This study is the first to report the average amount of care patients chose to pursue for their low back pain after a four week trial of either chiropractic care (flexion distraction) or physical therapy (active exercise) and to assess group differences.

## Methods

### Participants

Consecutive new patients with chronic low back pain were recruited from two chiropractic clinics and two allopathic clinics in a major metropolitan area. Additional recruitment efforts included media advertising such as radio and newspaper advertisements, press releases, cable television advertisements, local posters, and a local electronic sign advertisement. Patients meeting the criteria viewed a three-minute video demonstrating treatments and assessments, and were presented with an Institutional Review Board (IRB) approved informed consent form. Participants enrolled in the study were at least 18 years old, had a primary complaint of low back pain for more than three months, and had no contraindications to manual therapy. A more thorough description of inclusion and exclusion criteria is presented in a previous publication along with the sample size analysis [20].

### Interventions

**Figure 1:**
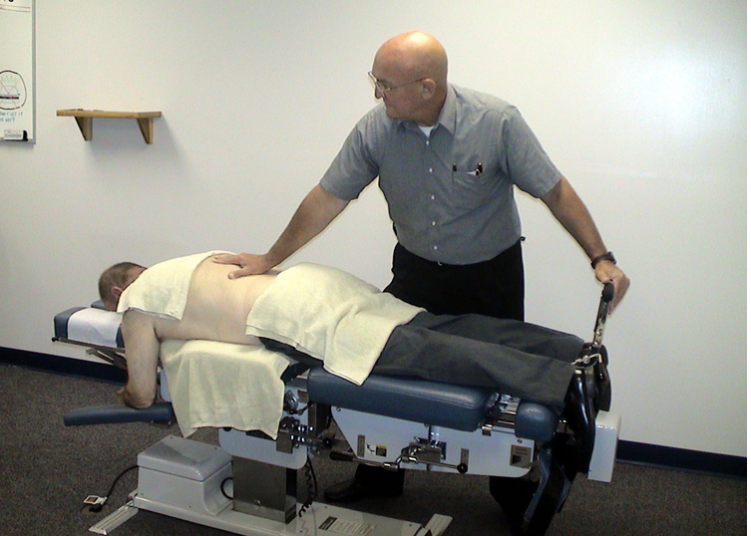


Participants were randomized to one of two forms of treatment. A random numbers table was used to develop the random assignment sequence, and each confidential random group assignment was placed in a consecutively numbered manila envelope by a Research Assistant not involved in this project. The two forms of treatment included: a series of flexion distraction procedures (FD) administered by chiropractors [21] and an active trunk exercise program (EP) administered by physical therapists. The FD technique was performed on a specially constructed table with a moveable headpiece, a stationary thoraco- lumbar piece, and a moveable lower extremity piece (see Figure 1). With the participant lying prone, the clinician placed one hand over the lumbar region at the level of interest and used the other hand to flex, laterally flex, and/or rotate the lower extremity section of the table. The FD intervention was administered by chiropractors with post-graduate certification in this technique. Application of treatment protocols was assessed and consistency between clinicians was confirmed by routine patient file checks.

**Figure 2:**
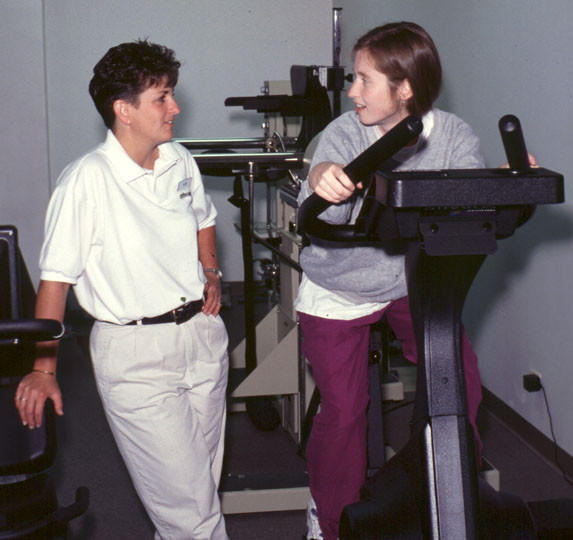


EP was administered by licensed physical therapists and consisted of strength exercises (see Figure 2), flexibility exercises, and cardiovascular exercises. Each participant receiving EP treatment followed a personalized program with type of exercise, amount of weight lifted, and number of repetitions based on their pain and disability levels. The aim of this program was to strengthen the muscles surrounding the spine and increase trunk flexibility. The physical therapists maintained treatment consistency through weekly group meetings.

Study participants in both study groups were treated for four weeks, two to four times per week at the discretion of the treatment provider. There was no significant difference in the number of treatments administered between the two treatment groups. Both groups received instructions for self-care, however consistency of providing this information was not collected. More information on these forms of care is located in a previous publication [20].

At the end of the four weeks of care, each participant was instructed that they were free to pursue any form of health care for low back pain, and that the purpose of the follow-up telephone calls were to track what forms of care (if any) were pursued.

### Objectives

The objective of this randomized clinical trial follow-up was to assess if there was a difference in the total number of office visits for low back pain over one year after a four week clinical trial of either physical therapy (EP) or chiropractic care (FD) for chronic low back pain. Our null hypothesis was that there were no group differences in the number of visits to any health care provider, to the chiropractor, or to the physical therapist.

### Outcomes

Health care utilization was measured on a weekly basis by a structured telephone interview during the year after active care. Weekly questions surrounded utilization of medical, alternative and complementary medicine, and self-care. The first section of the questionnaire asked whether or not participants attended a visit to any of the 16 possible health care providers (see Table 1), the number of visits that week, and whether or not the visit was for low back pain. The second section queried what forms of care were provided by the health care provider(s) such as medication, manipulation, etc. The final section of the questionnaire assessed use of self-care practices such as exercise, vitamins, or ergonomic changes not based on the advice of a health care provider. These data were secondary outcomes to the clinical trial pre and post-treatment pain and disability outcomes.

**Table 1:** Medical Providers Included in Assessment of Weekly Health Care Utilization

Acupuncturist
Chiropractor
Emergency Room
General Practitioner
Homeopath
Massage Therapist
Napropath
Neurologist
Nurse
Occupational Therapist
Orthopaedic Surgeon
Osteopath
Physical therapist
Psychiatrist
Psychologist
Rheumatologist
Other provider

### Analysis

Demographics and baseline characteristics of the two groups were compared using chi-square tests for categorical variables and t-tests for continuous measures. Groups of participants who did and did not withdraw from the study during the follow-up time period were similarly assessed for differences.

Descriptive data were calculated for the percent of participants receiving various forms of care for low back pain during the follow-up portion of the study, with Chi-square analysis determining group differences. Due to the scarcity of data, comparisons were only completed for the group difference in percent attending: (1) any health care provider listed in Table 1, (2) general practitioner/internist, (3) chiropractor, and (4) physical therapist.

The total number of visits to each type of health care provider was also calculated for each participant. Because some participants had missing data and other participants withdrew from study participation prior to study completion, the data set for each participant was annualized to extrapolate the expected number of visits if each participant had completed calls during every week of the follow-up year. For example, if a participant only responded to 26 weeks of calls (one half of the year) all data would be doubled to 'annualize' to a full year of data. Annualization was performed in lieu of missing data analysis due to the amount of unavailable data.

The annualized average numbers of visits per provider and median numbers were calculated; however because these numbers were of such low magnitude (typically close to 0), the ranges were also presented. Linear regression models were developed to assess the group difference in the annualized number of health care provider visits. Again, models were only created for the group difference in the annualized number of visits to: (1) any health care provider, (2) general practitioner/internist, (3) chiropractor, and (4) physical therapist. Because the data were not normally distributed, square root transformations were performed on the outcome variables prior to analysis. Covariates tested for significance were chosen based on expected influence of the outcome measures and included: (1) pain at the start of the follow-up period, (2) gender, (3) age, (4) presence of radiculopathy, and (5) presence of recurrent pain pattern.

Several forms of treatment were provided by health care providers, however the number and percent of participants who received only certain forms of treatments for low back pain were described, including: (1) over-the-counter medications, (2) prescription medications, (3) work sick leave, and (4) surgery.

Questions on self-care were included during each telephone interview. From this data, we calculated the number and percent of participants utilizing various forms of self-care for low back pain at any time during the year of follow-up. Major self-care categories were created by the investigators to better describe the data. Categories included (1) movement modification, (2) external application of treatment or a back support, (3) self medication, (4) dietary modification, (5) other changes to activities of daily living (ADL), and (6) alternative therapies including acupuncture, chiropractic, homeopathy, massage therapy, and napropathy. Major categories and individual items are presented as descriptive data only. All analyses were performed by using the Statistical Analysis System (SAS), Version 8.02 (SAS Institute, Inc., Cary, North Carolina).

## Results

### Participant flow

Recruitment of study participants began in August of 1998, was completed in December of 1999, and study participant follow-up was completed in February 2001. Numbers of people screened, reasons for exclusion, baseline demographics, and clinical characteristics are found in a previous publication [20].

Two-hundred and thirty-five participants were randomized into the study, 123 were allocated to FD and 112 to EP. Of the 235 participants randomized, 197 (83.8%) successfully completed the four-weeks of active care and agreed to begin the weekly phone calls.

### Numbers analyzed

Of the 197 participants who completed the active care within the study and agreed to participation in the follow-up portion of the study (83.8% of initial sample), six subsequently refused to participate in the weekly follow-up telephone calls. Therefore, a total of 191 participants initiated the weekly calls during the year of follow-up (81.3% of initial sample) with 107 participants from the FD group and 84 participants from the EP group.

Baseline characteristics and demographics were compared between groups and can be found in a previous publication [20]. The pain scores (VAS) were found to differ between the treatment groups at the start of follow-up (participants in the EP group had higher scores indicating significantly more pain). Therefore, the pain score at the start of follow-up were tested for significance in all models. Even though no other significant group difference was found, gender, age, presence of radiculopathy (pain in leg), and presence of recurrent pain pattern were also tested for significance within the analyses.

Of the 191 participants, 12 (6.3%) completed 1 to 13 calls, 4 (2.1%) completed 14 to 26 calls, 21 (11.0%) completed 27 to 39 calls, and 154 (80.6%) completed 40 to 52 calls. In terms of withdrawal, 13 FD participants and 25 EP participants withdrew from care prior to the follow up and 14 FD participants and 9 EP participants withdrew from the study during the follow up period. Groups of participants who did and did not withdraw from the study during the follow-up time period were assessed for differences, with the group who withdrew demonstrating an older age (by approximately 6.5 years). No other variable, including pain or disability, was associated with withdrawal from the study.

### Percent seeking care

Of the 191 participants followed, 41 (38%) of the FD participants and 45 (54%) of the EP participants sought care for low back pain from any provider during the year of follow-up, demonstrating a significant group difference (see Table 2). No group difference was noted in the percent of participants attending the general practitioner, the chiropractor, or the physical therapist, although a higher percent of participants sought general practitioner and chiropractic care in both groups compared to any other form of care. More participants in the EP group than the FD group sought care from specific health care professionals including: orthopedic surgeons and massage therapists. However, due to scarcity of data, these outcomes were not tested for statistical significance. Several providers who were listed on Table 1 were not included in Table 2 because no participants sought their care for low back pain during the year of follow-up.

**Table 2:** Number and Percent of Participants who Visited Specific Health Care Providers for Low Back Pain During One-Year Follow-Up

	FD group (n = 107)		EP group (n = 84)		
Outcome measure	N	%^a^	N	%^a^	P-value

Any health care provider	41	38.3%	45	53.6%	0.04*
					
General Practitioner/Internist	26	24.3%	28	33.3%	0.17
Chiropractor	13	12.2%	15	17.9%	0.27
Physical therapist	4	3.7%	4	4.8%	0.95
					
Emergency Room	3	2.8%	4	4.8%	
Orthopedic Surgeon	2	1.9%	8	9.5%	
Massage Therapist	1	0.9%	7	8.3%	
Acupuncturist	1	0.9%	2	2.4%	
Napropath	1	0.9%	1	1.2%	
Neurologist	1	0.9%	0	0%	
Psychiatrist	1	0.9%	0	0%	

^a ^Percentages of participants attending various health care providers may add up to more than 100% because some participants sought care from more than one provider.

* Treatment groups significantly different at p < 0.05

### Average number of visits sought

The numbers of visits to various health care providers demonstrated that, on average, participants in both treatment groups typically used very little medical care for low back pain (see Tables 3 and 4). Based on the upper ranges we note that some participants used quite a bit of care, for example one participant in the EP group visited the chiropractor 46 times during the follow-up year. Overall, participants mainly sought care from general practitioners/internists, chiropractors, and physical therapists. Some participants in the EP group also commonly visited the orthopedic surgeon, massage therapist, and acupuncturist. Annualized numbers of visits were similar to actual numbers of visits.

**Table 3:** Actual and Annualized Ranges of Numbers of Visits per Health Care Provider for Low Back Pain during One-Year Follow-Up

	FD group (n = 107)		EP group (n = 84)	
Outcome measure	Actual Ranges	Annualized Ranges	Actual Ranges	Annualized Ranges
Any health care provider	0 to 30	0 to 33	0 to 46	0 to 51
				
General Practitioner/Internist	0 to 6	0 to 7	0 to 10	0 to 15
Chiropractor	0 to 27	0 to 29	0 to 46	0 to 51
Physical Therapist	0 to 3	0 to 4	0 to 16	0 to 19
				
Emergency Room	0 to 2	0 to 3	0 to 1	0 to 1
Orthopedic Surgeon	0 to 5	0 to 6	0 to 6	0 to 15
Massage Therapist	0 to 3	0 to 3	0 to 11	0 to 12
Acupuncturist	0 to 3	0 to 4	0 to 12	0 to 13
Napropath	0 to 1	0 to 1	0 to 1	0 to 7
Neurologist	0 to 3	0 to 3	0 to 0	0 to 0
Psychiatrist	0 to 7	0 to 8	0 to 0	0 to 0

**Table 4:** Annualized Mean and Median Numbers of Visits per Health Care Provider for Low Back Pain during One-Year Follow-Up

	FD group (n = 107)		EP group (n = 84)			
Outcome measure	Mean	Median	Mean	Median	p-value^1^	p-value (non-parametric)

Any health care provider	2.2	0	6.0	1	0.005*	0.008**
						
General Practitioner/Internist	0.6	0	1.2	0	0.06	0.11
Chiropractor	0.9	0	2.7	0	0.13	0.21
Physical Therapist	0.1	0	0.3	0	0.92	0.97
						
Emergency Room	0.0	0	0.1	0		
Orthopedic Surgeon	0.1	0	0.4	0		
Massage Therapist	0.0	0	0.4	0		
Acupuncturist	0.0	0	0.2	0		
Napropath	0.0	0	0.1	0		
Neurologist	0.0	0	0.0	0		
Psychiatrist	0.1	0	0.0	0		

* Treatment groups significantly different at p < 0.05 using regression analysis

**Treatment groups significantly different at p < 0.05 using Wilcoxon-Mann-Whitney test for non-parametric variables

^1 ^Controlling for gender, presence of radiculopathy, and pain (VAS) at start of follow-up

Linear regression models were developed for the annualized number of visits to any provider, general practitioner, chiropractor, and physical therapist, and associated covariates were tested for significance within each model. There was a significantly lower number of office visits to any provider for low back pain by the FD group compared to the EP group during the year of follow-up (see Table 4). There was also a trend toward a lower number of office visits to general practitioners/internists by the FD group (p = 0.06). No group differences were demonstrated for the number of chiropractic or physical therapy visits during the year after care. Females, participants with radiculopathy, and participants with higher pain measures attended significantly more visits. All other potential confounders were found to be non-significant.

Because the data were non-normal, Wilcoxon-Mann-Whitney tests for non-parametric data were completed (see Table 4). The significantly lower number of office visits to any provider was again demonstrated in the FD group, however no group differences were noted when comparing the number of general practitioner, chiropractic, or physical therapy visits.

### Specific medical treatments

The percentage of participants who utilized specific medical treatments is presented descriptively. Of note, the amount and frequency of medication usage was not collected within this study.

As demonstrated in Table 5, the majority of participants within both groups took over-the-counter medications (77% FD, 87% EP), however only a minimal number of participants in both groups took prescription medications for back pain at some point during the year of follow-up (14% FD, 11% EP). Work sick leave occurred in 16% of participants in the FD group and 23% of the PT group. No known participants received surgery for low back pain during the year of follow-up.

**Table 5:** Results after 1-Year Follow-Up: Number and Percent of Participants Who Went on Sick Leave or Took Medication for Low Back Pain

	FD group (n = 107)		EP group (n = 84)	
Outcome measure	N	%	N	%

Took OTC medications for LBP	82	76.6%	73	86.9%
Took prescription medications for LBP	15	14.0%	9	10.7%
Took work sick leave due to LBP	17	15.9%	19	22.6%

### Self-care

Most participants (99% FD, 100% EP) used self-care (any form of care chosen by the patient that can be done on their own without physician support) at some point during the follow-up (see Table 6), with no apparent group difference in overall use of care. To better determine global patterns of care, we divided the types of self-care into six sub-categories. The most widely used type of self-care was movement modification (98% FD, 100% EP), with an almost equal number of participants increasing (56% FD, 61% EP) and decreasing/limiting (54% FD, 58% EP) their activities. The majority of participants who modified their movements indicated that they exercised at home (83% FD, 95% EP) and/or lifted differently (65% FD, 77% EP). More EP participants paid for help with house or yard work due to back pain (16% FD, 38% EP).

**Table 6:** Percent of Participants Using a Variety of Self Help Treatments

Self help treatment	FD group: # using (%) (n = 107)	EP group:# using (%) (n = 84)
Any self help measure	106 (99.1%)	84 (100.0%)
Movement modification	105 (98.1%)	84 (100%)
Ergonomic computer item	26 (24.3%)	18 (21.4%)
Exercise gym	59 (55.1%)	53 (63.1%)
Exercise home	89 (83.2%)	80 (95.2%)
Lift differently	69 (64.5%)	65 (77.4%)
Limit activity	58 (54.2%)	49 (58.3%)
Increase activity	60 (56.1%)	51 (60.7%)
Change activity	40 (37.4%)	33 (39.3%)
Cancel activity	25 (23.4%)	26 (31.0%)
Pay for house/yard work	17 (15.9%)	32 (38.1%)
External application/support	92 (86.0%)	75 (89.3%)
Back brace	30 (28.0%)	22 (26.2%)
Back support	40 (37.4%)	36 (42.9%)
Cane	9 (8.4%)	7 (8.3%)
Cold	37 (34.6%)	36 (42.9%)
Cream	34 (31.8%)	44 (52.4%)
Heat	75 (70.1%)	63 (75.0%)
Inversion boots	3 (2.8%)	3 (3.6%)
Orthotic	22 (20.6%)	25 (29.8%)
Self medication	88 (82.2%)	76 (90.5%)
Over-the-counter	82 (76.6%)	73 (86.9%)
Supplements	51 (47.7%)	54 (64.3%)
Dietary modification	54 (50.5%)	50 (59.5%)
Diet change	26 (24.3%)	24 (28.6%)
Increase water intake	47 (43.9%)	44 (52.4%)
Increase alcohol intake	11 (10.3%)	14 (16.7%)
Other activity changes	71 (66.4%)	61 (72.6%)
Change type of clothes	17 (15.9%)	17 (20.2%)
Sleep differently	66 (61.7%)	57 (67.9%)
Change/quit job	10 (9.4%)	7 (8.3%)
Use handicap car sticker	1 (0.9%)	1 (1.2%)
Alternative therapy	69 (64.5%)	65 (64.3%)
Biofeedback	4 (3.7%)	3 (3.6%)
Crystals	0 (0%)	1 (1.2%)
Magnet	9 (8.4%)	8 (9.5%)
Massage	62 (57.9%)	46 (54.8%)
Meditation	5 (4.7%)	12 (14.3%)
Reflexology	3 (2.8%)	2 (2.4%)
Self hypnosis	2 (1.9%)	4 (4.8%)

The second most common type of self-care utilized was external application of treatment for the back or back support (86% FD, 89% EP). Heat therapy for back pain was by far the most commonly used external application (70% FD, 75% EP), whereas cold therapy was less frequently used (35% FD, 43% EP). Back supports (37% FD, 43% EP) were more commonly used than back braces (28% FD, 26% EP), and creams for back pain were used more commonly by the EP group (52%) then the FD group (32%).

As previously discussed, self medication such as over-the-counter medications were commonly used (77% FD, 87% EP), as were supplements (48% FD, 64% EP).

Dietary modifications were somewhat popular (51% FD, 60% EP), with an increase in water intake being most common (44% FD, 52% EP) and diet changes being less common (24% FD, 29% EP). Interestingly, we found several participants who admitted to increasing their alcohol intake because of their low back pain (10% FD, 17% EP).

Changes in activities of daily living (66% FD, 73% EP), other than those already discussed, included many participants sleeping differently (62% FD, 69% EP) and changing the type of clothes they wore due to back pain (16% FD, 20% EP). Almost one-tenth of participants in both groups changed or quit their jobs due to back pain (9% FD, 8% EP).

Alternative therapies were popular (65% FD, 64% EP), although the majority of participants who utilized alternative therapies used massage therapy (58% FD, 55% EP) rather than the other methods of care. Magnet therapy was equally used in both groups (8% FD, 10% EP), whereas more EP participants used meditation (14.3%) than FD participants (5%).

## Discussion

The objective of this study was to report if there was a difference in the number of health care visits between a chiropractic treatment group (flexion distraction) and a physical therapy treatment group (exercise program) during the year after clinical trial care for low back pain. We found a significantly higher percent of EP participants compared to FD attending the office of any health care provider. We also found significant group differences in the number of visits, with the EP group attending a significantly higher number of visits to any provider. There did not appear to be any group differences in self-care habits, however we did note that most participants did three things: modified their movement (increased or decreased their activities), applied external therapies or back supports, and self medicated.

We hypothesized that there would be no group difference in the average number of visits to any health care provider. The results demonstrated that actually there were significant group differences during the year after trial participation, with a higher number of visits to any health care provider and to a general practitioner in the EP group. These results are the first to assess this difference, and a future focus on this issue is encouraged because number of visits relates to (1) the continued pain or disability after a clinical trial is complete and (2) added cost beyond that incurred within the trial. Maetzel and Li stated that "the cost of illness of low back pain is high and is comparable to other disorders such as headache, heart disease, depression, or diabetes" [22]. In this study, we did not track costs of care, although we did see that some participants utilized a great deal of care and that the costs of self-care could potentially be very high.

The self-care results in this study indicate that nearly every back pain participant utilized some form of self-care. Major differences include higher utilization rates in the EP group for exercising at home; paying for yard/house work; and utilizing back creams, over the counter medications, and supplementations; and meditation practices. Future studies may inquire if such self-care was utilized as back pain treatments or if such care was to prevent further back pain.

Several factors may have affected the results of this study. First of all, missing data was an issue which was attempted to be resolved by annualization of the data. However, annualizing the data does not necessarily reflect the actual amount of health care used by the participants, rather it reflects the approximate use of care based on the dates in which utilization data were collected. In future studies, self-report data could be compared with insurance records to verify health care provider types and numbers of visits. However, verification of self-report of self care is a challenge.

A second factor possibly affecting the results was that the type of practitioner visited and the number of visits is strongly dictated by insurance coverage, a factor we did not measure in this study. We expect that the coverage within this study was equal among groups based on the random nature of the study, however this information was not verified. Similarly, we did not collect data on co-pay amount, limitations to access of care, or re-injury; all issues which may have affected the amount of care utilized during the year post-care.

A third possible bias is that only participants who completed the treatments within the clinical trial were followed after care and included in this study. Participants who did not complete the trial may have chosen different courses of care than the participants who did complete the study, creating a sampling bias within our assessed population. We did not follow any participant who did not complete participation within the clinical trial so were not able to compare the follow up data between the participants who did and did not complete the study.

A final potential limiting factor for consideration in this and the associated publication is that a number of statistical tests have been performed on these data which increases the chance of type 1 error.

This was the first study to assess the amount of care utilized after a trial period of chiropractic care (flexion distraction) or physical therapy (exercise program). Investigators pursuing future studies that assess the health care utilization after trial care are encouraged to verify the amount of care received during the follow-up period.

## Conclusion

Based on one-year follow-up data imputed for complete analysis, participants who received physical therapy (exercise program) during a clinical trial attended a higher number of visits to any health care provider and to general practitioners during the year after care when compared to participants who received chiropractic care (flexion distraction) within the trial. Further studies are needed to verify these data.

## Competing interests

The author(s) declare that they have no competing interests.

## Authors' contributions

JC was the project manager for this study, completed the analysis, and drafted the manuscript. RG was the Principal Investigator on this study, initiated the project, secured funding, and assisted in manuscript writing. MM was the methodologist, assisted throughout the study and during manuscript preparation. JJ was the supervisor for treating chiropractors during the study and critically reviewed the manuscript. MK was the supervisor for physical therapists and was involved from the conception of the study, developed the protocols of physical therapy for the study, and critically reviewed the manuscript. AG was the medical co-investigator on this project from conception and critically reviewed the manuscript. AP was the biomechanical co-investigator on this project from conception and critically reviewed the manuscript. SE was the major thesis advisor of JC and oversaw analysis and manuscript preparation, and critically reviewed the manuscript.
